# Genetic reduction of chronic muscle pain in mice lacking calcium/calmodulin-stimulated adenylyl cyclases

**DOI:** 10.1186/1744-8069-2-7

**Published:** 2006-02-17

**Authors:** Kunjumon I Vadakkan, Hansen Wang, Shanelle W Ko, Evelyn Zastepa, Michele J Petrovic, Kathleen A Sluka, Min Zhuo

**Affiliations:** 1 Department of Physiology, Faculty of Medicine, University of Toronto Centre for the Study of Pain, University of Toronto, ON, Canada; 2 Graduate Program in Physical Therapy and Rehabilitation Sciences, University of Iowa, Iowa, IA, USA

## Abstract

**Background:**

The Ca^2+^/calmodulin-stimulated adenylyl cyclase (AC) isoforms AC1 and AC8, couple NMDA receptor activation to cAMP signaling pathways in neurons and are important for development, learning and memory, drug addiction and persistent pain. AC1 and AC8 in the anterior cingulate cortex (ACC) and the spinal cord were previously shown to be important in subcutaneous inflammatory pain. Muscle pain is different from cutaneous pain in its characteristics as well as conducting fibers. Therefore, we conducted the present work to test the role of AC1 and AC8 in both acute persistent and chronic muscle pain.

**Results:**

Using an acute persistent inflammatory muscle pain model, we found that the behavioral nociceptive responses of both the late phase of acute muscle pain and the chronic muscle inflammatory pain were significantly reduced in *AC1 *knockout (KO) and *AC1&8 *double knockout (DKO) mice. Activation of other adenylyl cyclases in these KO mice by microinjection of forskolin into the ACC or spinal cord, but not into the peripheral tissue, rescued the behavioral nociceptive responses. Additionally, intra-peritoneal injection of an AC1 inhibitor significantly reduced behavioral responses in both acute persistent and chronic muscle pain.

**Conclusion:**

The results of the present study demonstrate that neuronal Ca^2+^/calmodulin-stimulated adenylyl cyclases in the ACC and spinal cord are important for both late acute persistent and chronic inflammatory muscle pain.

## Background

In neurons, activity dependent cAMP synthesis is primarily mediated by membrane bound Ca^2+^/calmodulin-stimulated adenylyl cyclases (ACs). So far, ten members of the adenylyl cyclase family have been identified [1]. Of these, AC 1–9 isoforms are present in the brain (reviews, [2,3]). Two of the adenylyl cyclases, AC1 and AC8, are activated by calcium through the calcium-binding protein calmodulin [3]. These enzymes link activity-dependent increases in intracellular calcium to the production of intracellular cAMP. In addition, these neuronal adenylyl cyclases are viewed as coincidence detectors due to their specific interaction with NMDA receptors and voltage-dependent calcium channels at the neuronal membrane.

The role of the cAMP pathway in neurons along the pain pathway, including the anterior cingulate cortex (ACC) and the spinal dorsal horn were demonstrated [4,5] and discussed [6,7]. Peripheral injuries activate a series of signaling molecules downstream of adenylyl cyclases in neuronal populations. These molecules include pMAPK [8,9], transcription factor pCREB [10-12], and the immediate early genes *Egr-1 *[13,14] and *Arc *[15]. Neuronal adenylyl cyclases were shown to contribute to NMDA receptor-dependent synaptic potentiation in the hippocampus [16,17]. Recently, we showed that Ca^2+^/calmodulin-stimulated adenylyl cyclases are required to trigger synaptic potentiation in the ACC neurons [18] and synaptic facilitation in the spinal cord dorsal horn [19].

Chronic myofascial pain represents a considerable health problem [20]. Muscle pain is a symptom of various disorders including fibromyalgia, metabolic myopathies, myositis and also a side effect of medications, especially the statin group of cholesterol lowering drugs [21,22]. Injury to the muscle results in diffuse aching pain that is difficult to localize [23], spreading to regions outside the area of innervation [24]. Inflammation induced by intramuscular injection of capsaicin or carrageenan results in long-lasting (over weeks) bilateral secondary mechanical hyperalgesia. On the other hand, injection of these agents into the skin causes only a short-lasting, unilateral secondary mechanical hyperalgesia [25,26]. Although the role of adenylyl cyclases was shown to be important in behavioral sensitization associated with chronic subcutaneous inflammation and neuropathic pain [5,7,27], their role in inflammatory muscle pain has not been studied.

In the present work, we adapted a chronic inflammatory muscle pain model [28] and modified it to induce acute muscle pain by intramuscular injection of formalin. To overcome the non-selective action of adenylyl cyclase inhibitors (e.g. SQ 22536) used in previous studies [25,27], the present investigation used *AC1 *knockout (KO), *AC8 *KO and *AC1&8 *double knockout (DKO) mice to study the behavioral nociceptive changes after inducing acute persistent pain (formalin injection) and chronic pain (carrageenan injection) in comparison to the wild-type mice.

## Results

### Effects of deletion of AC1 and AC8 on motor function

**Figure 1:**
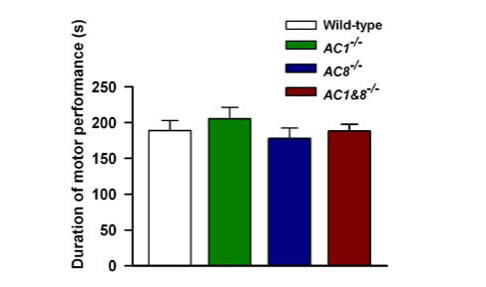
**Test of motor function in wild-type and *AC *KO mice**. Graph showing the latent period to fall from the accelerating RotaRod. There was no significant difference between wild-type, *AC1 *KO, *AC8 *KO, and *AC1&8 *DKO mice for the latency to fall, demonstrating that the motor function is intact. *n *= 5 mice in each group; *p *> 0.05.

To test whether deletion of *AC1*, *AC8 *or *AC1&8 *has any deleterious effect on muscle function, we tested their performance on an accelerating RotaRod. This test is widely used to assess motor ability in rodents by allowing them to walk on a rotating drum. Our studies indicated that *AC1 *and *AC8 *KO mice do not have any significant difference as compared to wild-type mice in their latency to fall from the RotaRod (*n *= 5 mice in each group; *p *> 0.05; Fig. 1). To test the possibility of compensation of function in these single KO mice, we performed the experiment on *AC1&8 *DKO mice. Similar to single KO mice, there was no significant difference between wild-type and DKO mice, indicating that the absence of AC1, AC8 or a combination of both isoforms does not affect baseline motor coordination.

### Role of AC1 and AC8 on acute persistent inflammatory muscle pain

**Figure 2:**
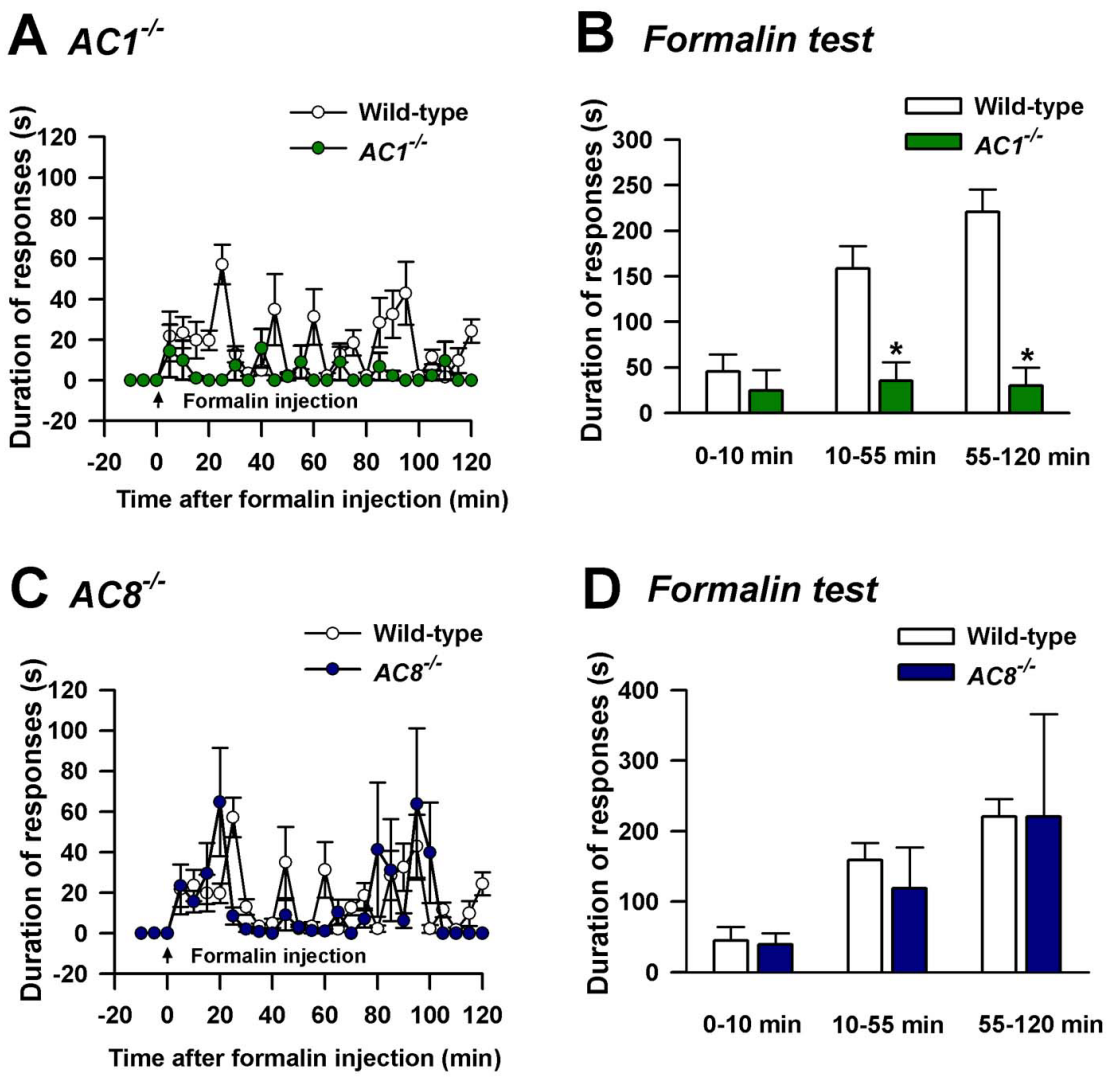
**Behavioral nociceptive responses to intramuscular formalin in *AC1 *and *AC8 *single KO mice**. Cumulative response for each 5 minutes time is plotted on y axis as duration of responses. A. Comparison of nociceptive responses to intramuscular formalin in wild-type and *AC1 *KO mice. Wild-type, *n *= 4 mice; *AC1 *KO, *n *= 8 mice. B. Data from experiment A is grouped as early, intermediate and late phases in comparison with wild-type mice. *p *< 0.05. *Significant difference from wild-type mice. C. Comparison of nociceptive responses to intramuscular formalin in wild-type and *AC8 *KO mice. Wild-type, *n *= 4 mice; *AC8 *KO, *n *= 8 mice. D. Data from experiment C is grouped as early, intermediate and late phases in comparison with wild-type mice.

**Figure 3:**
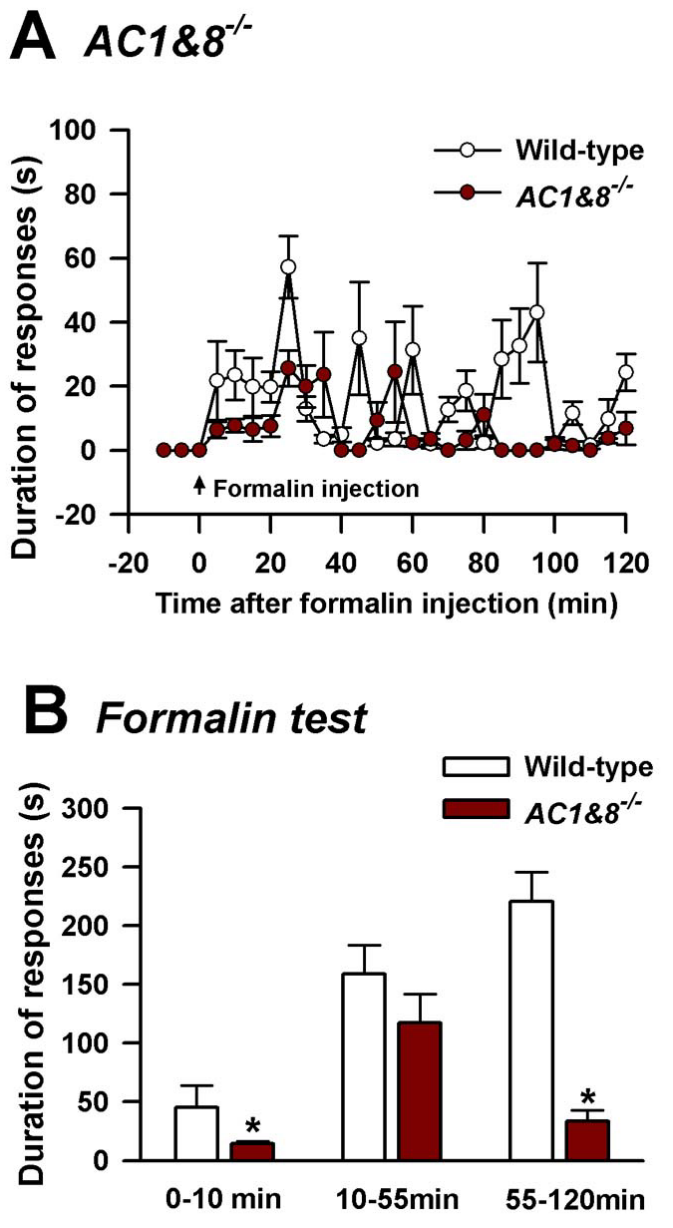
**Behavioral responses to intramuscular formalin in *AC1&8 *DKO in comparison with wild-type mice**. Cumulative response for each 5 min time is plotted on y axis as duration of responses. A. Comparison of nociceptive responses to intramuscular formalin in wild-type and *AC1&8 *DKO mice. Wild-type, *n *= 4 mice; *AC1&8*DKO, *n *= 8 mice; B. Data from experiment A is grouped as early, intermediate and late phases in comparison with wild-type mice. *p *< 0.05. *Significant difference from wild-type mice.

The contribution of AC1 and AC8 to the behavioral responses to acute muscle pain induced by intramuscular injection of 10 μL of 5% formalin (in normal saline) was examined. The behavioral responses were measured immediately after the injection. The formalin test is a common test of tissue injury-mediated inflammatory pain [5,29,30]. Licking and biting responses of the animal on the injected leg were observed for 120 min (wild-type *n *= 4 mice; *AC1 *KO, *n *= 8 mice, *AC8 *KO, *n *= 8 mice, *p *< 0.05) (Fig. 2A, 2C). Formalin-induced behavioral responses consisted of three phases [5]. The first phase consisted of the first 10 min after the injection in which behavioral responses were not significantly altered in *AC1 *KO or *AC8 *KO mice (Fig. 2B, 2D), indicating that AC1 and AC8 do not significantly contribute to the early phase of acute sensory responses to formalin independently. However, *AC1&8 *DKO mice showed significant reduction in the first phase. A significant difference was observed between wild-type and *AC1 *KO mice in phase 2, (*n *= 8 mice, *p *< 0.05) indicating the specific role of this Ca^2+^/calmodulin-stimulated adenylyl cyclase during the intermediate stage of nociception. In the final stage, behavioral responses of *AC1 *KO mice were significantly reduced compared to wild-type mice. By contrast, *AC8 *KO mice did not show significant behavioral changes in any of the phases compared to wild-type mice. In *AC1&8 *DKO mice, we found significantly reduced responses to formalin in phase three of behavioral responses (*n *= 8 mice, *p *< 0.05; Fig. 3A). These differences in responses in the different phases among *AC1 *KO, *AC8 *KO and *AC1&8 *DKO mice are possibly due to different molecular requirements involved in different stages of acute persistent inflammatory muscle pain.

### Chronic inflammatory muscle pain

**Figure 4:**
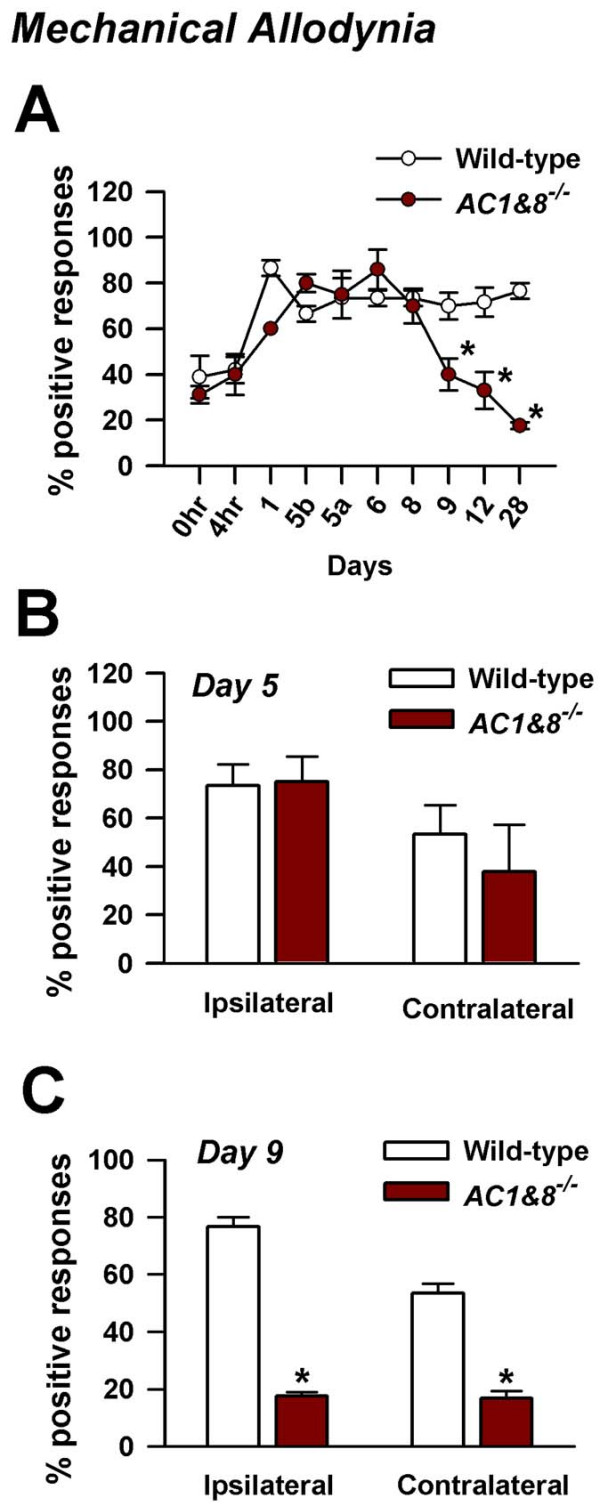
**Mechanical allodynia in chronic inflammatory muscle pain**. Intramuscular injections of carrageenan were given on day 1 and 5. The data is plotted as percentage of responses out of 10 mechanical stimuli over the dorsum of the foot at an interval of 5 min. Wild-type, *n *= 5 mice; *AC1&8 *DKO, *n *= 8 mice. A. The behavioral responses of animals to a non-noxious mechanical stimulus were recorded on the ipsilateral side. 5b denotes time point before second injection of carrageenan on day 5; 5a denotes time point after second injection of carrageenan on day 5. B. Mechanical allodynia on day 5 following the second intramuscular injection of carrageenan. There was no statistical difference between the wild-type and DKO mice on both ipsilateral and contralateral sides. C. Mechanical allodynia on day 9. Significant differences in mechanical allodynia were present between the wild-type and DKO mice on both ipsilateral and contralateral sides. *p *< 0.05. *Significant difference from wild-type mice.

To test the role of adenylyl cyclases in chronic inflammatory pain, we used *AC1&8 *DKO mice. The pain was induced by injecting 20 μL of carrageenan into the left gastrocnemius muscle, a protocol that was modified based on similar studies in the rat [26]. Injections were given on day 1 and day 5. Hind paw withdrawal to non-noxious mechanical pressure by a 2.44 mN von Frey fiber was measured before and at different time points after injection (wild-type, *n *= 5 mice, *AC1&8 *DKO, *n *= 8 mice) (Fig. 4A). The withdrawal response to mechanical force was increased 4 hours after the first carrageenan intramuscular injection. During the early phase and up to day 5, behavioral responses were not statistically different between wild-type and *AC1&8 *DKO mice (*p *> 0.05; Fig. 4B). On the following days, there was a gradual decrease in mechanical allodynia in *AC1&8 *DKO. These KO mice showed a statistically significant reduction in mechanical allodynia on the ipsilateral side by day 9 (*p *< 0.05; Fig. 4C). This significant reduction in mechanical allodynia was observed bilaterally on day 12 and remained reduced until the end of the experiment on day 28. This indicates that AC1 and 8 are involved in maintaining long lasting allodynia and in activating signal transduction pathways that lead to long lasting chronic muscle pain.

### Inhibition of nociceptive responses in acute and chronic inflammatory muscle pain by the novel AC1 inhibitor

**Figure 5:**
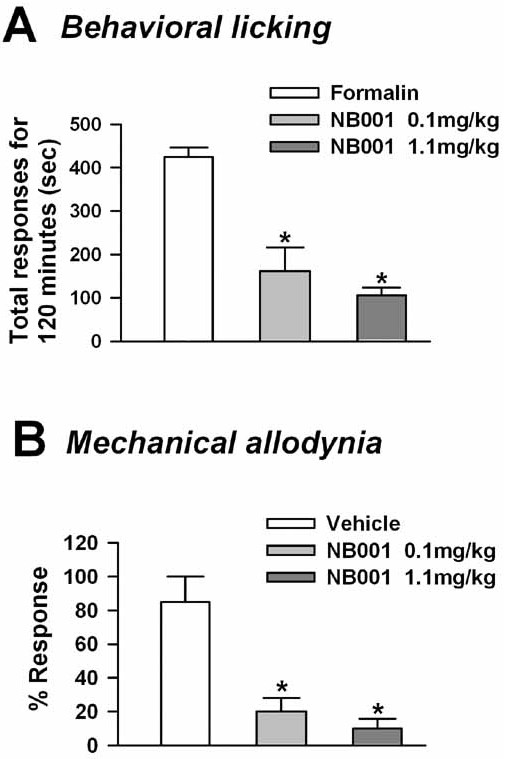
**Effect of novel AC1 inhibitor in acute persistent and chronic muscle inflammation**. A. Novel AC1 inhibitor NB001 produced significant reduction of licking responses in the late phase of the formalin test with peak response at 1 mg/kg body weight. *n *= 6 mice;*p *< 0.05. *Significant difference from vehicle. B. Effect of AC1 inhibitor NB001 on mechanical allodynia after chronic muscle inflammation. *n *= 4 mice; *p *< 0.05. *Significant difference from vehicle.

AC1 is more sensitive to calcium than AC8 [3]. Therefore, we decided to study the effect of blocking AC1 in both acute and chronic muscle pain. A novel AC1 inhibitor NB001 produced a significant reduction in behavioral responses to acute muscle inflammatory pain at doses ranging from 0.1 to 5 mg/kg body weight with a peak response at 1 mg/kg body weight (*n *= 6 mice; *p *< 0.001; Fig. 5A). The initial phase of the licking response during the first ten minutes was not changed after blockade of AC1 with NB001, similar to that observed in *AC1 *KO mice and as reported previously [5]. The total responses were reduced to almost one fourth, indicating that the AC1 inhibitor, at a concentration of 1 mg/kg body weight, is sufficient to block AC1 activity. The effect of the same AC1 inhibitor was also tested on chronic inflammatory muscle pain. NB001 significantly reduced mechanical allodynia at doses ranging from 0.1 to 1 mg/kg (*n *= 4 mice; *p *< 0.05; Fig. 5B); 1 mg/kg maximally reduced the mechanical allodynia, similar to that in acute inflammatory pain responses.

### Activation of non-Ca^2+^/calmodulin-stimulated adenylyl cyclases in the ACC 'rescued' behavioral sensitization

**Figure 6:**
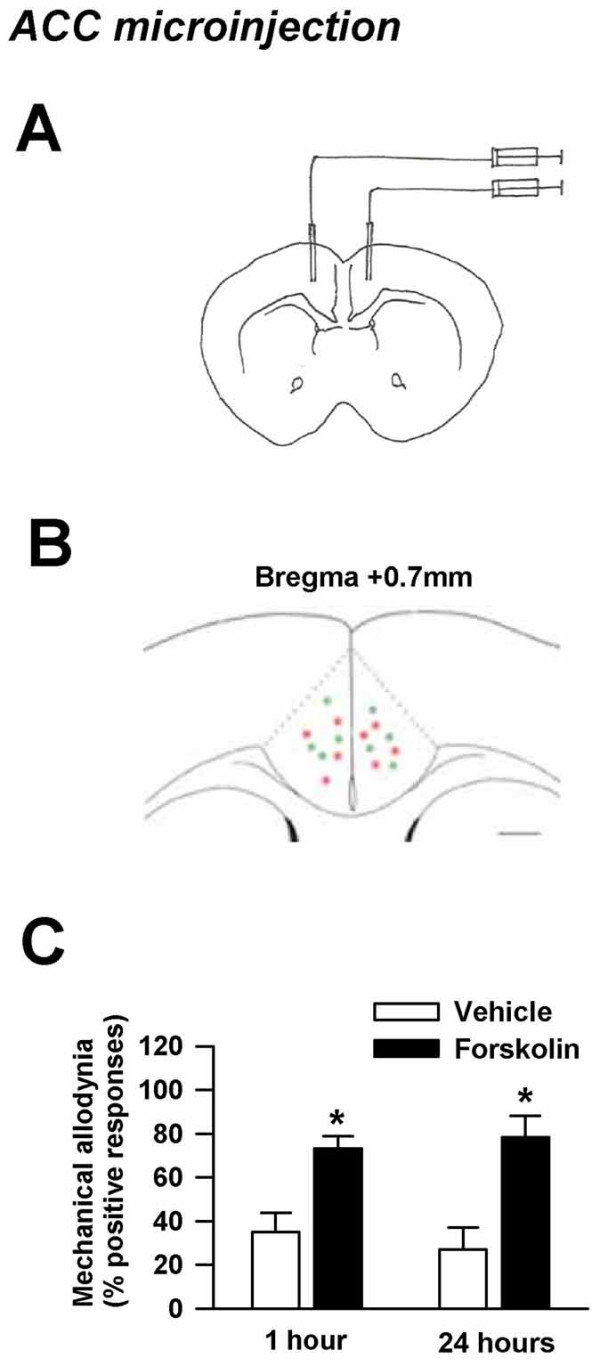
**Rescue of mechanical allodynia by bilateral injection of forskolin into the ACC**. A. Site of bilateral injection of forskolin into the ACC in coronal plane. Injections were made at 0.7 mm anterior to the bregma, 0.3 mm lateral to the midline and 1.75 mm ventral to the skull. B. Bilateral ACC microinjection sites of vehicle (in green) and forskolin (in red). The coronal section is 0.7 mm anterior to the bregma. C. Forskolin rescued behavioral allodynia in carrageenan induced chronic muscle pain in *AC1&8 *DKO mice on day 9 on the ipsilateral side. Forskolin injection, *n *= 6 mice; Vehicle injection, *n *= 5 mice. *p *< 0.05. *Significant difference from vehicle injection.

To examine whether activation of other adenylyl cyclases rescues the mechanical allodynia phenotype seen in the carrageenan muscle pain model in *AC1&8 *DKO mice, we injected forskolin (a non-specific adenylyl cyclase activator) into either the ACC, spinal cord or muscle on day 9 when mechanical allodynia was significantly reduced. The dose of forskolin was determined based on similar injections into the hippocampus and ACC in previous studies [5]. We performed microinjection of forskolin (12 nmolar solution, 0.5 μl) into both sides of the ACC of *AC1&8 *DKO mice (forskolin injection, *n *= 6 mice; vehicle injection, *n *= 4 mice; Fig. 6A, B). Mechanical allodynia was measured 1 hour after forskolin injection. Microinjection of forskolin bilaterally into the ACC produced mechanical allodynia ipsilaterally one hour after injection and remained elevated for 24 hours (Fig. 6C) (forskolin injection, *n *= 6 mice; vehicle injection, *n *= 4 mice; *p *< 0.05). The data suggests that cAMP produced by forskolin through the activation of other adenylyl cyclases in *AC1&8 *DKO can rescue mechanical allodynia. This finding, thus excludes the possibility that behavioral defects in DKO are due to developmental changes.

### Activation of adenylyl cyclases in the spinal cord, but not in peripheral tissue, rescued mechanical allodynia

**Figure 7:**
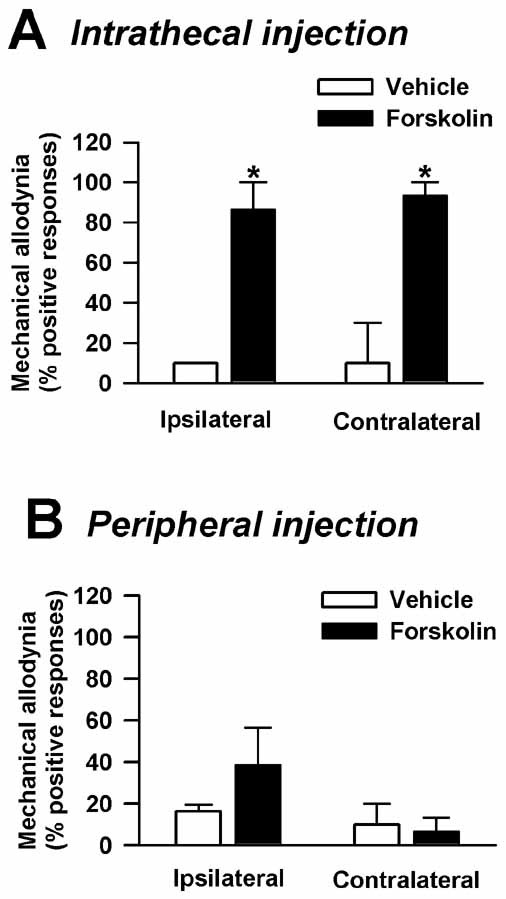
**Effect of spinal intrathecal and intramuscular forskolin on behavioral allodynia in chronic inflammatory muscle pain in *AC1&8 *DKO mice**. A. Intrathecal injection of forskolin rescued mechanical allodynia. Forskolin injection, *n *= 4 mice; Vehicle injection, *n *= 3 mice. *p *< 0.05. *Significant difference from vehicle injection. B. Intramuscular injection of forskolin did not rescue mechanical allodynia. Forskolin injection, *n *= 4 mice; Vehicle injection, *n *= 3 mice. *p *> 0.05

In addition to the ACC, the dorsal horn of the spinal cord is also important in regulating the threshold for mechanical allodynia. Intrathecal injection of forskolin in *AC1&8 *DKO mice produced mechanical allodynia bilaterally (Fig. 7A) (forskolin injection, *n *= 4 mice; vehicle injection, *n *= 3 mice; *p *< 0.05). This indicated that activation of adenylyl cyclases in the spinal cord by forskolin produced sufficient cAMP to activate the downstream cascade, rescuing the mechanical allodynia. To test whether there is similar activation of adenylyl cyclases at the peripheral nerve endings in the muscle, we injected forskolin into the gastrocnemius muscle, where muscle injections were originally given. This did not produce any significant effect on mechanical allodynia (Fig. 7B) (forskolin injection, *n *= 4 mice; vehicle injection,*n *= 3 mice; *p *> 0.05) indicating that peripheral activation of adenylyl cyclases, either within the nerve endings or in the non-neuronal cells, to increase cAMP has no effect in reducing the threshold of mechanical allodynia.

## Discussion

The present study examined the role of Ca^2+^/calmodulin-stimulated adenylyl cyclases AC1 and AC8 in both acute persistent and chronic muscle pain. AC1 and AC8 act downstream from NMDA receptors and contribute to NMDA receptor-dependent synaptic potentiation lasting several hours [16]. Our results provide genetic, pharmacological and behavioral evidence that adenylyl cyclase isoforms AC1 and AC8 are important for behavioral sensitization after muscle inflammation. Previous studies have examined the role of AC1 and AC8 in behavioral allodynia in subcutaneous inflammation [5]. Since the characteristics of pain arising from the muscle inflammation is different from that of cutaneous pain [23,24], we investigated the role of Ca^2+^/calmodulin-stimulated adenylyl cyclases in both acute persistent and chronic muscle inflammation. The behavioral responses during the early phase, after intramuscular formalin injection, were not significantly reduced either in *AC1 *or in *AC8 *single KO mice compared to the wild-type (Fig. 2B &2D). The behavioral responses during the late phase were significantly reduced in *AC1 *KO as well as *AC1&8 *DKO indicating that AC1 has direct contribution towards the late phase of acute persistent muscle pain.

### Adenylyl cyclases and synaptic enhancement

Ca^2+^/calmodulin-stimulated ACs are activated by NMDA receptor-mediated calcium entry and the reduction in the late phase of the formalin lick test may be due to the loss of NMDA receptor-dependent synaptic potentiation that normally lasts for several hours [16]. Our recent study has demonstrated that Ca^2+^/calmodulin-stimulated adenylyl cyclases are required for triggering NMDA receptor dependent synaptic potentiation in ACC neurons [18]. Therefore, one possibility is that the absence of Ca^2+^/calmodulin-stimulated adenylyl cyclases in KO mice prevents NMDA-dependent synaptic potentiation and leads to reduction in behavioral responses in the late phase of the formalin lick test. The activation of intra and/or extra-neuronal adenylyl cyclases in the ACC rescued mechanical allodynia. While the reason for this is not yet known, we hypothesize that direct activation of non-Ca^2+^/calmodulin-stimulated adenylyl cyclases may initiate cellular changes in neuronal and/or non-neuronal cells ultimately triggering downstream pathways following the cAMP step in neurons. In the spinal cord, the role of Ca^2+^/calmodulin-stimulated adenylyl cyclases is different. cAMP generated by adenylyl cyclase stimulated by forskolin acts synergistically with 5 HT to recruit functional AMPA receptors and provides a mechanism for long-lasting synaptic enhancement [19]. Therefore, activation of adenylyl cyclases by forskolin at the spinal cord is also a contributory mechanism for the phenotypic rescue in *AC1&8 *DKO mice after intrathecal injection.

### Role of adenylyl cyclases in maintaining chronicity of mechanical allodynia

Chronic inflammation caused by carrageenan induced a sensitization process (allodynia) in normal animals. Repeated injections of carrageenan on days 1 and 5 were sufficient to maintain this allodynia up to day 28. In DKO mice, nociceptive responses were reduced by day 9. While adenylyl cyclases are required for maintaining the late phases, it cannot be ruled out that initiation of the activity by these Ca^2+^/calmodulin-stimulated adenylyl cyclases in the early phase is required for activation of downstream cascades that maintains the late phases. The cAMP synthesis induced by nociceptive responses during persistent pain may have a dose-dependent effect on a downstream cascade that regulates adenylyl cyclase synthesis and activity. In addition, coupling of calcium and cAMP systems may result in an ordered activation or a positive feedback regulation of calcium and cAMP dependent protein kinases and possibly provide positive feedback regulation of calcium channels, thus maintaining mechanical allodynia. Elevated cAMP signals arising from activation of AC1 and 8 by calcium may therefore play an important role in synaptic plasticity associated with chronic muscle pain.

The present study demonstrates that Ca^2+^/calmodulin-stimulated adenylyl cyclases AC1 and 8 induce a sensitized state in the nociceptive pathway and provides evidence that they are critical in the maintenance of muscle inflammatory pain. Specifically, the role of highly calcium sensitive AC1 is critical as evidenced by the significant reduction of behavioral nociceptive responses in the late phase of both acute and chronic muscle pain following intra-peritoneal injection of the AC1 inhibitor. However, in animals with genetic deletion of AC1 and AC8, forskolin may rescue behavioral phenotypes by activating non-Ca^2+^/calmodulin-stimulated adenylyl cyclases in both the ACC and spinal cord. These results indicate that cAMP signaling in both the ACC and spinal cord are important for the maintenance of chronic muscle inflammatory pain. Taken together, the results of our previous report [5] and the present study consistently suggest that Ca^2+^/calmodulin-stimulated neuronal adenylyl cyclases play a key role in mediating the late phase of acute persistent as well as chronic inflammatory pain.

## Methods

### Animals

Adult (8–12 weeks) male mice lacking adenylyl cyclase isoforms AC1, AC8 and both AC1 and AC8 [5,16,31] were maintained on a C57BL/6 background and were crossed back at least 12 generations. Adult (8–12 weeks) C57BL/6 mice were used as controls. KO mice were well groomed and did not show any signs of congenital abnormalities or motor coordination defects. Experimenters were blind to the genotype. Animals were maintained on a 12 hour light dark cycle with food and water available *ad libitum*. The experimental protocols were approved by the Animal Care Committee at the University of Toronto.

### Motor function test

Motor function was tested using an accelerating RotaRod (Med Associates). One hour before testing, animals were trained on the RotaRod at a constant acceleration of 16 rpm until they could remain on for a 30-sec period. The RotaRod test was performed by measuring the time each animal was able to maintain its balance walking on the rotating drum. The RotaRod accelerated from 4 to 40 rpm over a 5-min period. Mice were given three trials with a maximum time of 300 sec and a 5 min inter-trial rest interval. The latency to fall was taken as a measure of motor function. Some mice hold on to the rotating drum instead of walking. For these mice, the latency to fall was recorded after 2 complete rotations.

### Formalin lick test for acute inflammatory muscle pain

Formalin (10 μL; 5% in normal saline) was injected deep into the gastrocnemius muscle on the left side. The needle was directed from the lateral side to avoid any bone penetration and the tip was stopped at the middle of the muscle for injection. The total time spent licking or biting the injected left leg, including the thigh and paw, was recorded every 5 min for 2 hours immediately following the injection. Responses from 0 to 10 min were plotted as early nociceptive responses, those from 10 to 55 min as intermediate, and those from 55 to 120 min as late nociceptive responses.

### Chronic inflammatory muscle pain model

Mice were briefly anaesthetized with isoflurane. 20 μL of carrageenan (3%, in normal saline pH 7.2, 20 μL) or normal saline control (pH 7.2) was injected into the left gastrocnemius muscle, as described in the formalin lick test. This protocol was adapted from similar experiments that induced chronic muscle pain in rats [26]. Care was taken during the injections due to the high viscosity of carrageenan. The injection site was mildly massaged to ensure proper diffusion of the drug from the injection site. To induce chronic muscle pain, two intramuscular injections of carrageenan were administered on days 1 and 5.

### Measurement of mechanical allodynia

Mice were allowed to acclimatize to the chamber for 30 min before testing. A threshold stimulus was determined by an animal's paw withdrawal upon application of a von Frey filament (Stoelting, Wood Dale, Illinois) over the dorsum of the left hind paw to the point of bending. Mechanical sensitivity of the animal to the innocuous pressure of a 0.4 mN von Frey filament (No.2.44) was scored and repeated every 5 min for up to 10 times. Positive responses included prolonged hind paw withdrawal followed by licking or scratching and these responses were plotted as percentage positive responses. Injections were carried out on days 1 and 5. Mechanical allodynia was tested on days 1, 2, 5 (before and after injection), 6, 8, 9, 12 and 28. For rescue experiments with forskolin, mechanical allodynia was tested thirty minutes after forskolin microinjection. A two-way analysis of variance (ANOVA) was used to compare differences.

### Intraperitoneal injection of AC1 inhibitor

The novel AC1 inhibitor NB001 was dissolved in 1% DMSO in normal saline and injected into the peritoneal cavity in doses varying from 0.1 to 5 mg/kg body weight in a volume of 10 μL/gram body weight. The effect of the drug was tested 30 minutes after the injection.

### ACC injections

After anesthetizing with 2% halothane (30% O_2 _balanced with nitrogen), mice were placed in a Kopf stereotaxic instrument. A microinjection apparatus, consisting of a Hamilton syringe (50 μL) connected to an injector needle (30 gauge) by a thin polyethylene tube and a motorized syringe pump (Razel Scientific Instruments Inc., Stamford, Connecticut), was used to perfuse the drugs. The skin over the scalp was incised at the midline and bilateral openings were made in the skull to allow the insertion of a microinjection needle into the ACC. The coordinates of the injection were as follows: 0.7 mm anterior to Bregma, 0.3 mm lateral to the midline, and 1.75 mm ventral to the surface of the skull [32]. Either 0.5 μl forskolin (12 nmolar) or a vehicle solution (20% DMSO in filter-sterilized phosphate-buffered saline, pH 7.4) was infused at a rate of 0.05 μl/min. The needle was withdrawn 5 min after completion of the injection. Upon completion of experiments, all animals were deeply anesthetized and perfused transcardially with saline, followed by 4% paraformaldehyde. Serial cryostat coronal sections (30 μm) of the ACC were mounted on glass slides, counterstained with hematoxylin and examined under the microscope to confirm the site of injection. All chemicals were purchased from Sigma (St. Louis, MO).

### Spinal intrathecal injections

After anesthetizing with 2% halothane with 30% O_2 _balanced with nitrogen, mice were placed in a Kopf stereotaxic instrument. The anesthesia set-up was the same as that used for the ACC microinjection. The lumbar vertebrae were slightly flexed, spines were palpated and the needle was advanced manually through the inter-spinal space lateral to the spinous processes as described [33]. Either 0.5 μl forskolin (12 nmolar) or a vehicle solution (20% DMSO in filter-sterilized phosphate-buffered saline, pH 7.4) was infused at a rate of 0.05 μl/min. After the completion of the injection, the needle was kept in place for 5 min before the withdrawal.

### Data Analysis

Results are expressed as mean ± standard error of the mean (SEM). Statistical comparisons were performed by a two-way ANOVA. *p *< 0.05 was considered statistically significant. For the RotaRod test, data were analyzed using a one-way ANOVA.

## Authors' contributions

KIV carried out majority of the work, wrote the manuscript, prepared the figures.

HW carried out the tests for the effect of NB001 drug on muscle pain.

SWK standardized the protocols for allodynia and rescue experiments.

EZ participated in allodynia measurements.

MJP carried out the RotaRod test.

KAS collaborated with this work.

MZ designed and supervised the work.
